# Tissue classification by rapid evaporative ionization mass spectrometry (REIMS): comparison between a diathermic knife and CO_2_ laser sampling on classification performance

**DOI:** 10.1007/s00216-019-02148-8

**Published:** 2019-11-11

**Authors:** Michele Genangeli, Ron M. A. Heeren, Tiffany Porta Siegel

**Affiliations:** 1 Maastricht MultiModal Molecular Imaging (M4I) Institute, Division of Imaging Mass Spectrometry, Maastricht University, Universiteitssingel 50, 6229ER Maastricht, The Netherlands; 2 School of Pharmacy, Chemistry Unit, University of Camerino, Via S. Agostino 1, 62032 Camerino, MC Italy

**Keywords:** Bioanalytical methods, Laser ablation, Tissue analysis, Lipids, REIMS, Mass spectrometry

## Abstract

The increasing need for rapid, in situ, and robust tissue profiling approaches in the context of intraoperative diagnostics has led to the development of a large number of ambient ionization-based surface sampling strategies. This paper compares the performances of a diathermic knife and a CO_2_ laser handpiece, both clinically approved, coupled to a rapid evaporative ionization mass spectrometry (REIMS) source for quasi-instantaneous tissue classification. Several fresh meat samples (muscle, liver, bone, bone marrow, cartilage, skin, fat) were obtained from different animals. Overall, the laser produced cleaner cuts and more reproducible and higher spectral quality signals when compared with the diathermic knife (CV laser = 9–12%, CV diathermic = 14–23%). The molecular profiles were subsequently entered into a database and PCA/LDA classification/prediction models were built to assess if the data generated with one sampling modality can be employed to classify the data generated with the other handpiece. We demonstrate that the correct classification rate of the models increases (+ 25%) with the introduction of a model based on peak lists that are tissue-specific and common to the two handpieces, compared with considering solely the whole molecular profile. This renders it possible to use a unique and universal database for quasi-instantaneous tissue recognition which would provide similar classification results independent of the handpiece used. Furthermore, the laser was able to generate aerosols rich in lipids from hard tissues such as bone, bone marrow, and cartilage. Combined, these results demonstrate that REIMS is a valuable and versatile tool for instantaneous identification/classification of hard tissue and coupling to different aerosol-generating handpieces expands its field of application.

**Electronic supplementary material:**

The online version of this article (10.1007/s00216-019-02148-8) contains supplementary material, which is available to authorized users.

**Figure:**
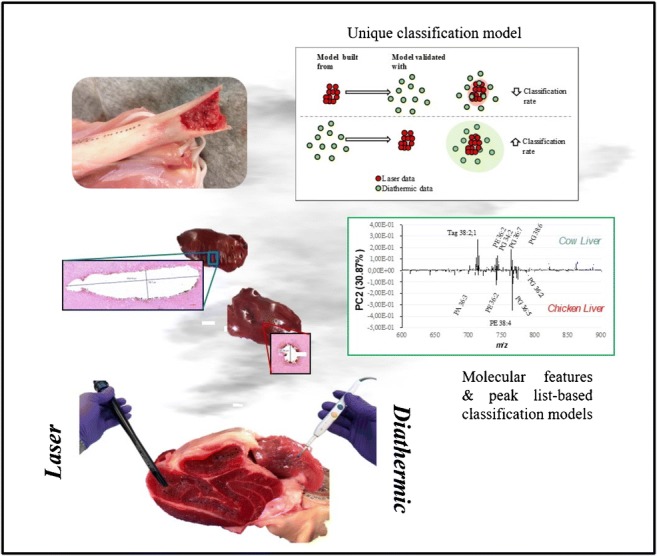
Graphical abstract

## Introduction

Accurate tumor diagnosis during surgery solely relies on histopathological evaluation of frozen section and is still challenging. Surgeons would benefit from instantaneous feedback during surgery to improve tumor resection and ultimately patient outcome. The need for more precise oncological surgical procedures depends on the availability of rapid, in situ, and robust molecular approaches. This has led to the development of a large number of ambient ionization mass spectrometry (MS)–based surface sampling strategies [1–4]. Amongst these ambient ionization techniques, desorption electrospray ionization (DESI), or laser ablation electrospray ionization (LAESI), to name a few, has been developed as robust solutions for lipidomic/metabolomic imaging and profiling [5, 6]. However, they are not suited for in situ rapid analysis of “fresh” or intact samples. Other sampling techniques such as the “MasSpec Pen” [7], “SpiderMass” [8, 9], and cavitron ultrasonic surgical aspiration/sonic spray ionization (CUSA/SSI) [10] have been hyphenated to ambient ionization MS to enable rapid intraoperative diagnostics of human cancers. The MasSpec Pen makes use of a simple and disposable hand device which allows for non-tissue-destructive sampling. However, Saudemont et al. recently reported that the MasSpec Pen could have limited use for in vivo cancer diagnostics due to possible contamination of the system with tissue debris, which is caused by the fact that the device has to be operated in liquid contact with the tissue [11]. The “SpiderMass” approach is based on a modified optical parametric oscillator (OPO) system pumped by an Nd:YAG infrared laser to superficially ablate tissue (micro invasiveness) for in vivo tissue classification [9]. Clinical implementation of these two techniques for intraoperative diagnostics remains limited because they have not yet been approved as a medical device. Most of the aforementioned techniques allow only the sampling of specimens under ambient conditions, and cannot be used to resect tissues. Woolman et al. reported the combination of DESI-MS and a picosecond infrared laser (PIRL) MS as an ablation probe requiring minimal tissue removal for ex vivo/in situ imaging operations [12]. They also suggested how the integration of PIRL with rapid evaporative ionization mass spectrometry (REIMS) is likely to increase the robustness of signal and reproducibility of PIRL due to the aspiration driven by the Venturi pump [13].

REIMS is an ambient ionization technique which involves the aspiration and rapid thermal ionization of aerosols produced from electrocautery, ultrasonic aspiration [10], and ultraviolet (UV) and infrared (IR) lasers [14–17]. The most common handpieces employed to generate smoke and combined with a REIMS system are monopolar and bipolar electrocautery/diathermic (blade/needle) knives routinely used in the operating room (OR) for surgical procedures [15]. A monopolar device uses a higher voltage when compared with a bipolar device and has a better ability to cut and coagulate large bleeding areas. Thus, a monopolar accessory is mainly used to cut and coagulate relatively large tissues while the bipolar electrocautery is more used for smaller tissue applications (e.g., brain surgery) or for the classification/identification of bacteria and fungi [18, 19].

Amongst other certified medical devices, a CO_2_ laser [20] can also be used intraoperatively due to (i) its infrared wavelength of 10.6 μm highly absorbed in water molecules contained in tissue [21–23] and (ii) its ability to produce both a high-power continuous wave (i.e., for cutting) and pulsed and super pulsed waves (i.e., for sampling [8, 13]). The strength of the approaches described above is the use of standard surgical devices that are already frequently employed in a surgical setting. No modifications of the surgical procedures are needed while surgeons are offered an opportunity to make better informed decisions. This facilitates the easier take-up and acceptance by the medical community.

REIMS was originally developed for oncological surgical applications [15, 24, 25] with the ultimate goal to improve patient outcome after surgery by ensuring the removal of all malignant tissue. The applicability of REIMS for cancerous tissue diagnostics has been demonstrated both ex vivo and in vivo in the operating room with instantaneous tissue classification (“normal”/surrounding tissue vs. tumor or tumor margins). The ex vivo procedure entails a surgical resection of biological material, followed rapidly by a REIMS-enabled assessment of the tissue outside the patient, while the in vivo studies are conducted during resection of the material from the patient.

REIMS has also been used for food analysis (e.g., to identify the species of origin of meat products) [26] and for identification of bacterial colonies and direct in vivo mapping of bacterial growth [27, 28]. The combination of REIMS with multivariate analysis of the data collected from the aerosols allows for classification of the dissected tissues within seconds [28]. Different statistical methods are employed to build a tissue recognition model based on tissue-specific molecular profiles. From the literature, principal component and linear discriminant analyses (PCA/LDA) are mainly reported for this purpose [4, 15, 24, 25, 28–30]. The entire molecular profile within the mass range *m/z* 600–900—which corresponds to the range of detection of glycerophospholipids—is used to build these models. Differences in the relative intensity of lipid species are included to maximize the variance between two or more groups. Lately, Cordero Hernandez et al. reported that the classification rate of tissues can be improved by selecting tissue-specific peaks and generating new models based on both PCA and LDA of acquired molecular profiles [31].

**Fig. 1:**
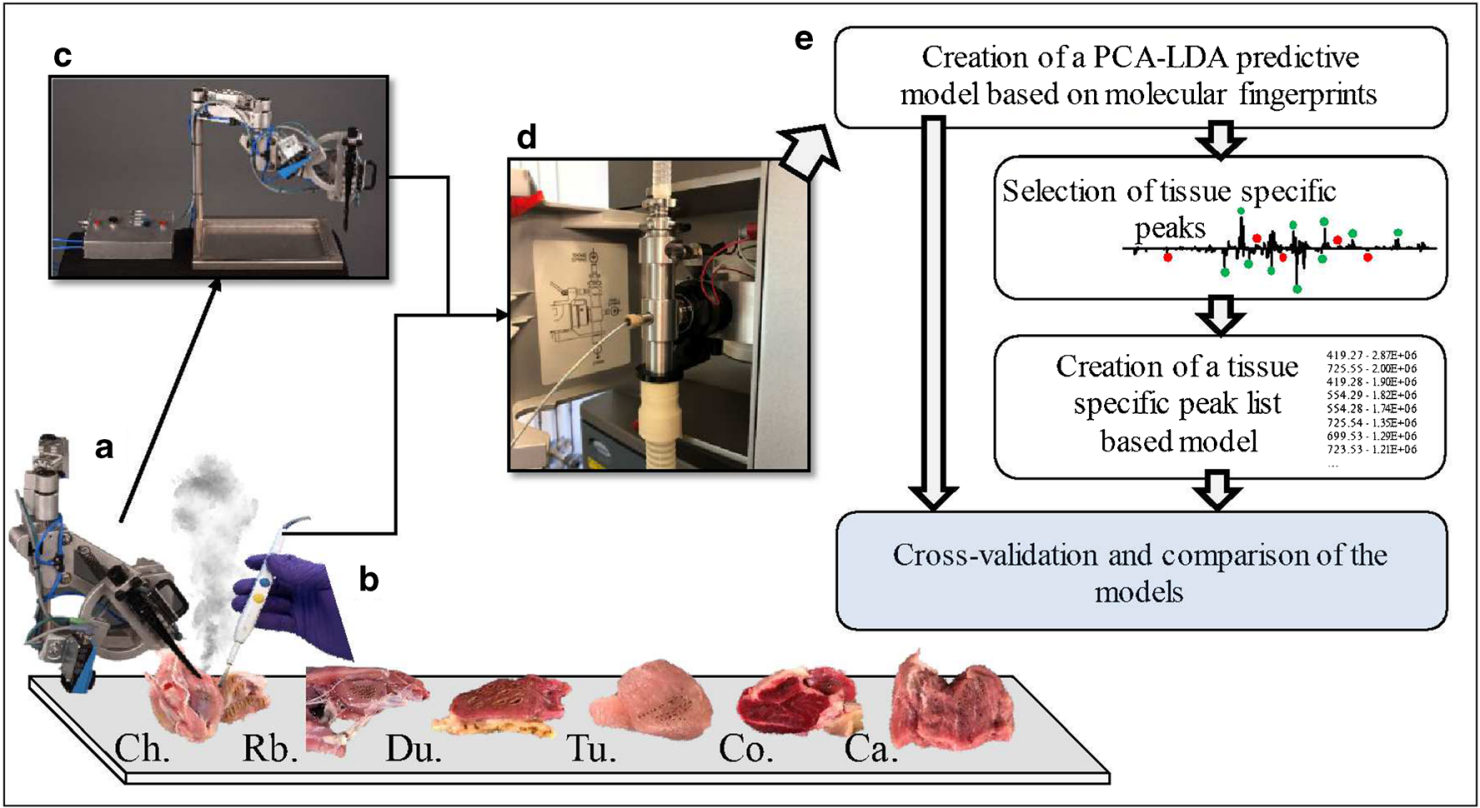
Experimental workflow from tissue sampling to the creation of tissue classification models: Every tissue (chicken [Ch.], rabbit [Rb.], duck [Du.], turkey [Tu.], cow [Co.], and calf [Ca.]) was sampled and analyzed with both the CO_2_ laser [**a**] and the electrocautery knife [**b**]. For safety reasons, the laser handpiece was mounted on a home-built mechanic arm [**c**] maintained in a fixed position during laser ablation. Both handpieces were connected to the REIMS source and the data collected from vapors were acquired in negative ionization mode [**d**]. After basic data preprocessing, several PCA-LDA predictive models were created based on the overall molecular (lipids) fingerprint [**e**]. From the loading plots of each model, tissue-specific peaks were selected to create peak lists common to the two handpieces. This peak list was further used to build a more targeted model to improve the classification rate of our statistical models that are now able to classify tissues independently from the handpiece used for sampling

Here, we report the benefits and limitations of two surgical handpieces: a CO_2_ laser and a diathermic knife to generate smoke from fresh animal tissues. These handpieces were coupled to a REIMS-quadrupole time of flight (qTOF) systems for quasi-instantaneous tissue classification. For this purpose, fresh meat samples (muscle, liver, bone, bone marrow, cartilage, skin, fat) were obtained from different animals and analyzed using the two sampling tools. Generated aerosols were directly analyzed via REIMS and tissue/species-specific molecular profiles were recorded. The handpieces were compared in terms of tissue damage/invasiveness, spectral quality, and signal-to-noise ratio. The molecular profiles were subsequently entered into a database and classification/prediction models based on PCA/LDA were built. The goal of our next investigations was to demonstrate whether it was possible to build a unique and universal database for quasi-instantaneous tissue recognition which would provide similar classification results independent from the handpiece used. Therefore, we compared the tissue classification rates and reproducibility of the following: (i) the PCA/LDA molecular classification models built first with data generated with one sampling modality and then employed to classify the data generated with the other handpiece; (ii) a more targeted approach where the models were built based on features that are common for both handpieces. The general workflow is reported in Fig. 1. We confirmed that the classification rate of the models increases with the introduction of model-based peak lists that are tissue-specific; which renders it possible to use one database for both handpieces. Finally, we demonstrate that the CO_2_ laser can be used as a sampling tool for hard tissues such as bone, cartilage, and bone marrow, expanding the field of applications and the versatility of REIMS for tissue classification.

## Materials and methods

### Materials and reagents

Isopropyl alcohol, ethanol, methanol, xylene, and water (ULC/MS-CC/SFC grade) were purchased from Biosolve Chimie (Dieuze, France). Norhamane was purchased from Merck KGaA (Darmstadt, Germany). Leucine-enkephalin was purchased from Sigma-Aldrich (St. Louis, USA).

### Diathermic knife

Electrosurgical dissection was carried out using a commercial monopolar electrosurgical unit COVIDIEN Force Fx electrosurgical generator (COVIDIEN Ltd., Ireland) providing power-controlled sinusoidal 330-kHz alternating current and a custom handpiece and active cutting electrode (blade length = 1.7 cm, width = 0.2 cm, thickness = 0.05 cm) modified by Waters (Manchester, UK) with a smoke evacuating line (tubing system). The generator was used in “cut” mode with a power setting range of 20 to 30 W. All the ex vivo samples were placed on a return electrode of the electrosurgical setup. The heater in the REIMS source was kept at 300 °C. In order to maximize reproducibility, the burns made with the diathermic knife were carried out while keeping the knife vertical to the tissue being analyzed, and the signal coming from the smoke was recorded for 3 s.

### Surgical CO_2_ laser

**Fig. 2:**
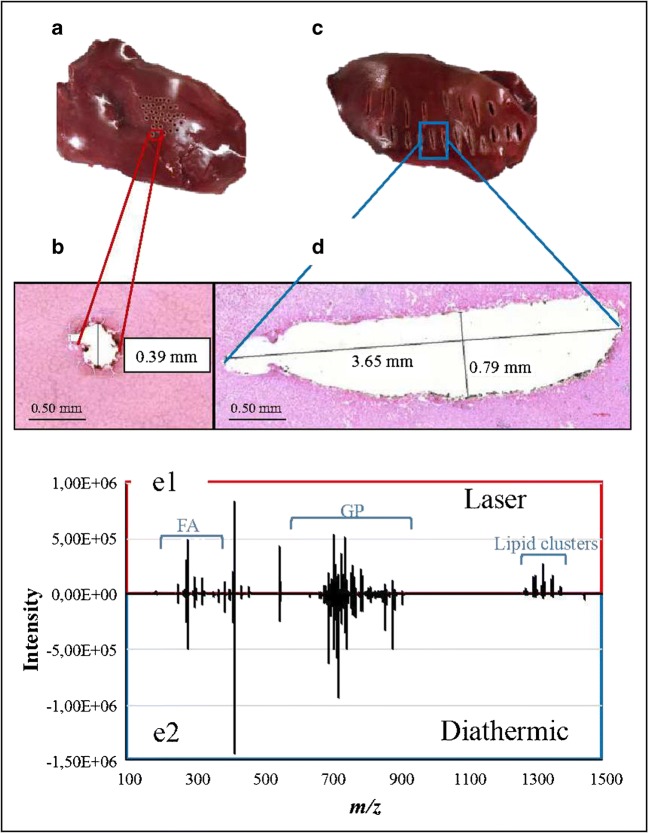
Macroscopic [**a**, **c**] and microscopic (H&E stained) [**b**, **d**] visualization of the tissue damages generated after laser [**a**, **b**] and diathermic [**c**, **d**] samplings. The laser generated a smaller tissue damage compared with the diathermic knife (laser ⌀ = 0.39 mm, diathermic ⌀ = 0.79 mm wide/3.65 mm long). The laser also produced a more reproducible signal, reduced spectral noise, and more lipid clusters (in the region *m*/*z* 1200–1500) [e1–e2]. FA, fatty acids; GP, glycerophospholipids

All experiments were performed with an AcuPulse Class IV CO_2_ 10.6-μm surgical laser unit (Lumenis GmbH, Germany) with a maximum power of 60 W. The laser can be operated in three distinct modalities (continuous wave [CW], pulsed wave [PW] and super pulsed wave [SPW] modes). Continuous wave mode produces a low peak power; it has a superficial “soft” impact and a wider thermal zone (spot size 1.3 mm, 170 mJ energy, 60% density, and 150–200 μm penetration depth; Fig. 2). The pulsed and super pulsed modalities have a high and very high peak power respectively, a deeper impact, and a narrow and controlled thermal zone (spot size 0.12 mm, 20 mJ energy, 15% density, and 700 μm penetration depth; Fig. 2) [32]. The laser was used in “cut” pulsed mode with a laser power ranging between 18 and 20 W (matched to the tissue being analyzed), using single pulse time of 0.15 s. For hard tissue analysis only, a higher power was necessary in pulsed mode to generate sufficient ions due to the relative low water content of the bone and cartilage. The laser was fixed to a home-built mechanic arm (see Electronic Supplementary Material (ESM) Fig. S1) for safety considerations. This consequently increased the reproducibility by keeping the laser at a fixed distance from the samples (focus point). The arm is made of aluminum grade 6082 (chemical-resistant, non-corrosive, and light material) and glass bead blasted on the outside (in order to reduce the reflectivity of the aluminum). Each moving part of the arm is kept steady by air-powered breaks. A visible-infrared camera (sick inspector PIM60) was mounted next to the laser handle in order to control the laser height from the tissue, keeping it at a fixed distance from the tissue, increasing the reproducibility of the data generated. The focus point was controlled by a homemade program which allows the laser to operate only when it is focused on the tissue.

### REIMS—qTOF instrumentation

The aerosols generated with the two handpieces were analyzed via REIMS. The smoke produced was aspirated by a Venturi pump connected to the REIMS source. The REIMS source was mounted on a qTOF mass spectrometer (G2-XS, Waters, Manchester, UK) operated in negative ionization mode. Data was acquired within the mass-to-charge range *m/z* of 100–1500 and in “sensitivity” mode, at a mass resolution of 15,000 full width at half maximum (FWHM) at *m/z* 600. The instrument was calibrated daily with a solution of sodium formate. A solution of leucine-enkephalin dissolved in isopropyl alcohol (1 ng/μl) was continuously infused in the REIMS source at a flow rate of 150 μl/min. The deprotonated ion of Leu-Enk was used for external lock mass calibration of the data acquired (*m/z* 554.2615 [M-H]^−^).

### Experimental design

Meat samples (muscle, liver, bone, bone marrow, cartilage, and skin) from different animals (cow, calf, chicken, turkey, duck, and rabbit) were obtained from a local butcher and analyzed consecutively and on the same day with the surgical laser and diathermic knife (Table 1). An average of *n* = 20 burns was recorded for every tissue with both handpieces (with the exception for bone and cartilage as noted in Table 1). The samples were frozen in liquid nitrogen after analysis with both handpieces and then sliced (12 μm thick) with a cryo-microtome (Leica CM 1860 UV, Leica Biosystems, Wetzlar, GE), mounted onto histological glass slides (VWR, Leuven, BE) for additional analysis with matrix-assisted laser desorption/ionization mass spectrometry imaging (MALDI-MSI).

**Table 1:** Number of burns collected for each tissue type and for each handpiece. Bone, bone marrow, and cartilage were analyzed only with the CO_2_ laser

Animal	Subtype	*n* burns Laser	*n* burns Diathermic
Chicken	Muscle	25	25
	Skin	28	20
	Liver	25	22
	Bone	30	–
	Cartilage	23	–
	Bone marrow	9	–
Rabbit	Muscle	25	20
Duck	Muscle	25	25
	Skin	15	15
Turkey	Muscle	20	20
Cow	Muscle	25	25
	Liver	25	25
Calf	Muscle	25	25

### MALDI-MSI experiments and lipid identification

The identification of selected lipid ion species was performed by both MS/MS fragmentation on the REIMS-qTOF and the exact mass measurement from MALDI-MSI on a Q-Exactive Orbitrap (Thermo Fisher Scientific, MA, USA). For the MALDI-MSI experiments, the matrix was sublimed on top of the sample as follows: a solution of 60 mg of norhamane and methanol (2 ml) was applied onto the slides with a sublimator device (HTX Technologies, LLC, USA; [33]). The REIMS-TOF was set to MS/MS negative ionization mode with variable collision energy (see details in the ESM). For the Orbitrap, the laser power was set to 1.6 μJ per pulse at 1 kHz and the resolution was set at 240,000 in negative ionization mode at *m/z* 200–2000. Alex123 and LIPID metabolite and Pathways Strategy Lipidomic Gateway (LIPIDMAPS®) were used for the lipid identification based on the exact mass and MS/MS data. The identification of selected peaks of interest was performed limiting the search to only deprotonated glycerophospholipid and fatty acid ions in the *m/z* range 600–900 with a mass tolerance of ± 0.1 Da for the REIMS and ± 0.002 Da for the Orbitrap (ESM Tables S1–S4).

### Hematoxylin and eosin histological staining

The hematoxylin and eosin (H&E) staining protocol used consisted of the following: (i) matrix removal from the plates with a 100% ethanol (2×) bath for 3 min; (ii) rehydration in subsequent ethanol baths of 96% (2×) and 70% (2×), each for 2 min; (iii) staining with hematoxylin for 3 min, washed in a deionized water bath; (iv) staining with eosin for 30 s; (v) final wash in water (3 min), ethanol (1 min), and xylene (30 s). Once dry, the slides were covered with a coverslip that was fixed with Entellan (Merk KGaA, Darmstadt, Germany). High-resolution optical images of the H&E-stained slides were acquired with a MIRAX Desk Scanner (Zeiss, Jena, Germany).

### Multivariate data analysis and building of the statistical models

Mass spectral processing and multivariate data analysis were performed using the Abstract Model Builder (AMX) software ([beta] version 1.0.1581.0, Waters Research Center, Budapest, Hungary). The mass spectra were background subtracted, mass drift corrected based on the reference peak at *m/z* 554.2615 [M-H]^−^ corresponding to the deprotonated ion of leucine-enkephalin used as lock mass. Peaks were binned to 0.1 Da within the mass range *m/z* 600–900. The spectra were all normalized against the total ion count (TIC). The resulting data was used to create two classification models: (i) a principal component analysis/linear discriminant analysis (PCA/LDA) model based only on molecular fingerprints which is used to identify tissue-specific peaks and (ii) a peak list–based PCA/LDA model. PCA was performed with a maximum of 10 dimensions and LDA with *n* − 1 dimensions where *n* is the number of tissue types introduced in the model. Several PCAs with an increasing number of components were tested and the results showed that with a maximum of 10 components, we describe > 98.8% of the total variance of the data. A series of internal cross-validation tests were performed by building the classification models from 80% of the data and validating it with the remaining 20%, or by validating a model generated with one sampling modality, such as the laser, with the data generated with the other modality, such as the diathermic knife, and vice versa. For all cross-validation, outliers were identified based on standard deviation (sd) as follows: when a feature deviates 5sd, it is considered as an outlier. The TIC of twenty consecutive burns (*n* = 10 for the diathermic knife and *n* = 10 for the laser) from every tissue type were compared and the results expressed in terms of coefficient of variation (CV). The average signal-to-noise ratio (S/N) from ten selected peaks over five consecutive burns was calculated and the average results were reported in terms of CV.

### Safety considerations

All samples were analyzed in a class II biosafety cabinet. All solvents used for the analysis and cleaning were handled according to the material safety data sheet provided by their respective manufacturer. In order to comply with the safety regulations of using a CO_2_ laser in our laboratory and increase the reproducibility of the data generated with the laser, a dedicated mechanic arm allowing the laser to be operated at fixed position was developed (see the “Surgical CO_2_ laser” section). The laser was fired through a two-button safety box placed outside the biosafety cabinet (ESM Fig. S1b), while wearing CO_2_ laser–grade safety goggles in a reserved area shielded with class IV safety curtains.

## Results

### Visual assessment of tissue damages and evaluation of spectral quality for real-time analysis of soft tissues

Here, we compare the main differences between the two handpieces (laser and diathermic knife) through a comparison of (i) tissue damage and handpiece characteristics; (ii) stability and reproducibility of the generated signals; and (iii) qualitative assessment of generated mass spectra. The same tissue samples were consecutively analyzed with the diathermic knife and laser in order to facilitate a direct comparison of the handpieces and limit instrumental variability.

#### Comparison of handpiece’s characteristics and tissue damage

The tissue damage caused by two handpieces were macro- and microscopically examined. Figure 2a/c shows a macroscopic image of cow liver samples burnt with the laser and diathermic knife. It clearly demonstrates that tissue damage caused by the laser is more confined and localized than damage cause by the diathermic knife. This is mainly due to the width of the laser beam which is smaller than the blade used with the diathermic knife. In order to confirm and illustrate these observations, the same tissue was frozen in liquid nitrogen, cut with a cryotome, and H&E stained. As shown in Fig. 2b/d, the laser produced tissue burns of ≈ 0.39-mm radius while the diathermic knife (equipped with a blade electrode) produced much bigger burns of ≈ width = 0.79 mm and length 3.65 mm. It was hypothesized that the minimal material ablated, and thus the reduced smoke generated by the laser, led to more precise sampling.

#### Signal stability and reproducibility

The laser allowed the generation of lipid-rich smoke with either the continuous wave, pulsed wave, or super pulsed wave modes (ESM Fig. S2) for either cutting or surface sampling, demonstrating its versatility. We obtained the best results in terms of overall signal intensity with a laser power of 18 W and 0.15-s burn time in pulsed mode for all the soft tissues. The diathermic knife generated a more intense signal from the lipid species with a power of 25 W in cut mode. We observed the following: the possibility to control the penetration power, wave type, and position of the laser in respect to the sample being analyzed led to cleaner spectra and more intense signal intensities overall compared with the diathermic knife. The total ion count (TIC) of *n* = 10 mass spectra (from muscle, from all animals, 10 for the laser and 10 for the diathermic handpiece) from consecutive burns revealed a higher burn-to-burn reproducibility for the laser with a coefficient of variation (CV) ranging between 9.76 and 12.00%. The CV of the signal measured with the diathermic knife for different burns was between 13.92 and 22.72% for different tissues. The background noise level was constant for all tissues when analyzed with the same handpiece. The diathermic knife showed a noise level 1.6 times higher than that with the laser. The S/N ratio for the laser was (calculated from 10 peaks of the same tissue analyzed with both handpieces) ≈ + 17 times higher than that for the diathermic knife (see ESM Table S5).

The laser generated 7% more peaks than the diathermic knife (see the next paragraph), and the intensity of 10 selected peaks through 5 consecutive burns in the same tissue was evaluated resulting in an average peak intensity CV of 0.76% for the laser and 1.94% for the diathermic knife (see ESM Table S5).

As previously mentioned, it is worth noting that during our laser experiments, the laser was kept in a fix position for safety consideration (see the Safety paragraph in the “Materials and methods” section) and operated using a fixed laser power and exposure time while the burns with the diathermic knife were manually performed (the burn depth and time were manually evaluated trying to keep a 3-s burn time and constant handpiece movement through the tissue). The higher reproducibility and the more stable TIC intensity from the laser when compared with the diathermic knife were mainly due to the fixed position and operation parameters of the laser, which allowed more controlled sampling. This statement is supported by a recent publication from Bodai et al. where it is reported that the geometry and position of the electrode also play a major role in the classification performances of predictive models when using REIMS [34].

#### Qualitative assessment of generated mass spectra

The laser generated a higher peak intensity at *m/z* 1200–1400 and lower intensity in the “fatty acid” (*m/z* 100–350) and glycerophospholipid/triglyceride (*m/z* 600–1100) regions of the spectra when compared with the diathermic knife (Fig. 2). MS/MS was performed to identify selected ion species in the mass range *m/z* 600–1100 and the peaks within *m/z* 1200–1400 using both the REIMS-qTOF equipped with the diathermic knife and the MALDI-Orbitrap. Selected peaks were isolated with an isolation window of ± 1 *m/z* and fragmented with the REIMS-qTOF. A high collision energy was required (> 40 eV) for the peaks in the high molecular weight range of the spectra (*m/z* 1200–1400). MS/MS for this last group of molecules revealed several glycerophospholipids in the range *m/z* 600–800 plus a series of fatty acids in the range *m/z* 100–350 (ESM Fig. S3). After the REIMS analysis, the same sample was frozen, cut into 10-μm-thick sections and analyzed with the MALDI-Orbitrap where high mass accuracy measurements were performed for identification of the precursor ions. No species in the range *m/z* 1200–1400 were detected in the MALDI-Orbitrap MS spectra (ESM Fig. S3).

The high fragmentation energy is required (i.e., 40 eV) and several lipid species visible in the fragmentation spectra during MS/MS suggest that the species detected with the range at *m/z* 1200–1400 are in fact lipid clusters. The lipid clusters were not visible when using MALDI, while they were present when using the CO_2_ laser (with a high intensity) and diathermic knife (lower intensity). The thermal ablation induced by the CO_2_ laser and diathermic knife caused the desorption of tissue aerosols that are more prone to the formation of clusters under ambient conditions. The absence of a matrix and ambient pressure (REIMS) also contributed to the formation of lipid clusters [35–37]. Furthermore, the high energy released from the diathermic knife caused either a higher fragmentation or reduced cluster formation during sampling in comparison with the laser [38–40]. A further discussion of the formation and behavior of the lipid clusters is beyond the scope of this study.

The laser overall generates a better S/N and a more stable and reproducible signal when compared with the diathermic knife. The laser was operated in a fixed position in a safety arm that prevents laser operation when operator hands are present in the measurement space. It was not possible to mount the diathermic knife onto the same mounting arm and in its current state, the safety arm is not motorized. As a result, it cannot raster or be automated for electrocautery applications. On the other hand, the laser promotes the formation of lipid clusters resulting in a lower intensity for species of interest such as glycerophospholipids (but yet higher S/N with respect to the same species analyzed with the diathermic knife due to reduced background signal).

### Development of universal and more targeted tissue classification

Our ultimate goal is to improve the overall performance of our classification models (i.e., accuracy, robustness, and classification rate) and develop a unique database to correctly identify tissues independently from the handpiece used for sampling. Several classification models were generated for this purpose with the data acquired with the CO_2_ laser and the diathermic knife independently. Subsequently, one model was applied to the other data set to assess versatility of the classification model. The first classification models generated were based on molecular patterns solely, which helped to identify specific features from tissue types and common to the two handpieces to build more targeted classification models. The cross-validations were performed by keeping the value for the standard deviation multiplier for the outliers equal to 5.

#### Classification models based on molecular fingerprints

**Fig. 3:**
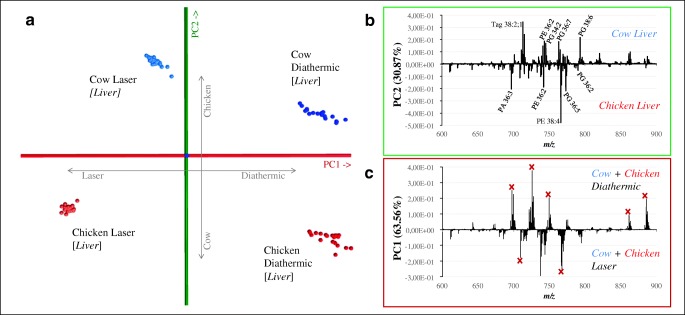
A PCA-LDA predictive model used for the creation of inclusion and exclusion peak lists. [**a**] Score plot from a model including cow liver and chicken liver analyzed with the two handpieces. From the loading plot, peaks able to discriminate between the two tissue types [**b**] were selected as an “inclusion list” (referred to as “Inc.”). Peaks able to discriminate between the two handpieces [**c**] were selected as an “exclusion list” (referred to as “Excl.”). The two peak lists were compared, and the peaks included in the exclusion list were removed from the inclusion list in order to create tissue-specific peak lists (tissue-specific peak list = Inc. − Excl.)

**Fig. 4:**
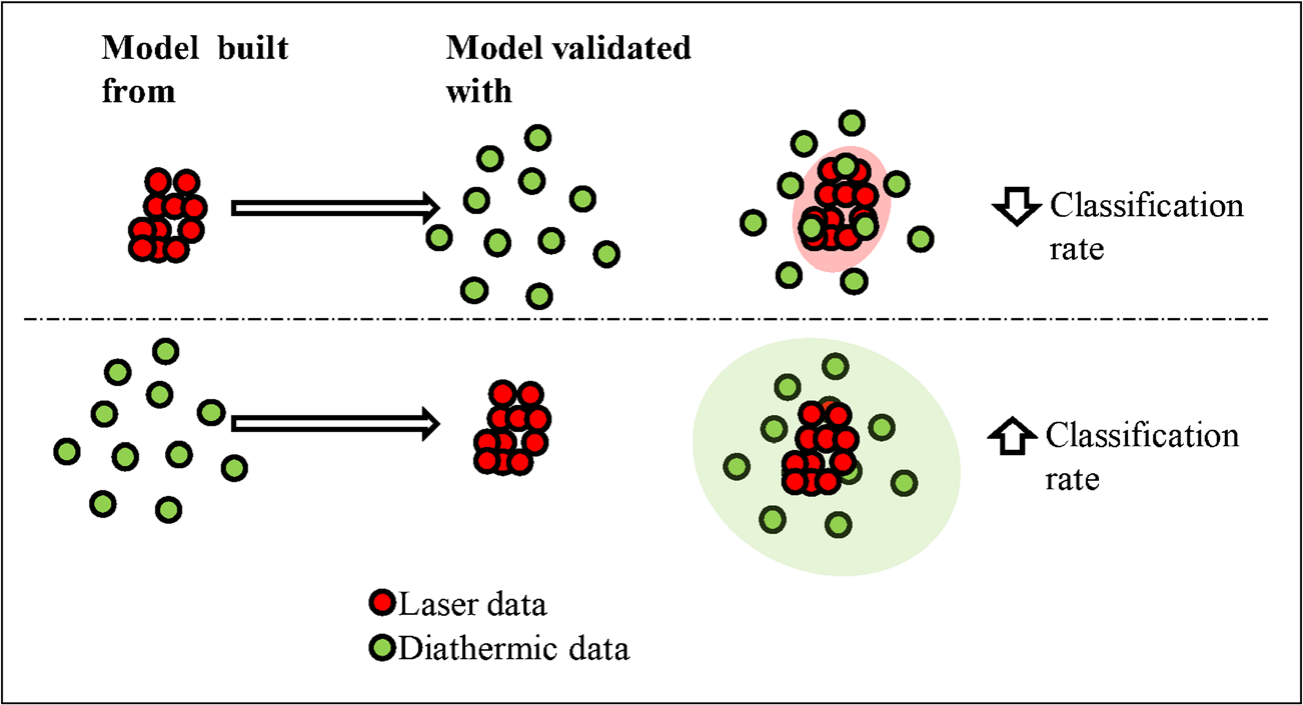
Schematic interpretation of the influence of the data variation in the creation of a predictive model. The data not included in the ellipse (represented by the light-colored ovals) are those which returned as outliers by the cross-validation process. This illustrates why the use of a more precise model leads to more outliers when classifying a subsequent dataset which inherently comprises more variance

Most tissue classification models are untargeted and based on molecular patterns within the detection range of lipid species (e.g., glycerophospholipids, triglycerides). Several classification models were created based on PCA/LDA—including either all the tissues or a subset of animal/tissue types were created (ESM Figs. S4–S6). This allowed the discrimination between tissues and the exclusion of outliers. The models were then subjected to a series of cross-validations where a model generated with the data from one handpiece was used to classify the data generated with the other handpiece, and vice versa. As reported in Table 2, the cross-validation using all tissue samples and animals show that it is possible to correctly classify only 58.64% of the tissue samples when using the diathermic knife–based classification/prediction model to classify data from the laser. The classification rate accuracy decreased to 28.73% when using the laser-based model to classify the data from the diathermic knife. The cross-validation using one tissue sample (either muscle or liver) from two animals led to a 100% classification rate for most of the tissues (with the exception for turkey/duck muscle) when using the diathermic knife–based classification/prediction model to classify data from the laser. This classification rate is halved (52%) when performing the cross-validation in the opposite manner (Table 2). Based on these results, building a model from the diathermic data and subsequently classifying the laser data provide a more accurate classification rate. A plausible explanation lies in the difference in precision and therefore variation between the data generated by the two handpieces (Figs. 3a and 4). The diathermic measurements are less precise, causing a higher variance in the predictive model (Fig. 4). This results in a lower chance to obtain outliers when classifying data from the laser and therefore in a higher score for the cross-validation with the laser data [41, 42]. Training a model from the data obtained with the laser creates a model with less variance which is more prone to generating outliers when cross-validating with less precise data (Fig. 4).

**Table 2:** Percentage of well-recognized tissue after cross-validation. The first column represents the tissue type/animal included in their respective model. The second and third columns represent the results of the cross-validation when the classification model was built with data generated with the diathermic knife and used to classify the data acquired with the laser. The fourth and fifth columns represent the results of the cross-validation when the classification model was built with data generated with the laser and used to classify the data acquired with the diathermic. *Mol. fin.*, classification model based on molecular fingerprints

	Cross-validation			
Data used to create the model	Diathermic knife		Laser	
Model used to classify the data acquired with:	Laser		Diathermic knife	
Animal/tissue included in the model	Mol. fin.–based model (%)	Peak list–based model (%)	Mol. fin.–based model (%)	Peak list–based model (%)
All**	58.640	70.890	28.730	55.830
All no skin	67.440	86.940	55.250	66.940
Chicken*	59.960	92.312	59.650	67.590
Chicken/duck (muscle)	100.000	90.310	50.000	52.940
Chicken/turkey (muscle)	100.000	100.000	51.350	84.360
Chicken/cow (liver)	100.000	100.000	52.630	78.250
Chicken/cow (muscle)	100.000	100.000	53.350	100.000
Chicken/calf (muscle)	100.000	100.000	52.470	100.000
Chicken/rabbit (muscle)	100.000	100.000	54.850	100.000
Cow*	77.780	98.450	52.680	76.390
Cow/calf (muscle)	100.000	100.000	52.120	100.000
Cow/rabbit (muscle)	100.000	100.000	51.540	100.000
Calf/rabbit (muscle)	100.000	100.000	52.110	100.000
Turkey/duck (muscle)	50.000	56.360	50.000	100.000
Turkey/cow (muscle)	100.000	100.000	51.130	100.000
Duck/cow (muscle)	100.000	100.000	52.140	100.000
Duck/rabbit (muscle)	100.000	100.000	53.960	100.000

**Muscle + liver from calf, chicken, cow, duck, rabbit, turkey

*Muscle + liver

We here recommend building unique classification models for the two handpieces on the diathermic data, only if the data is well segmented. The higher variance of the data from the diathermic knife could otherwise lead to misclassification of tissues. The classification performances of the models can be improved by introducing targeted features, as reported in the next paragraph.

#### Introduction of a targeted features to improve classification performance

An ideal situation with real-time tissue classification would be to discriminate different tissue types based on a classification database independently from the handpiece used. Models including tissue-specific peak lists have recently proven to lead to better classification rates [31]. Several classification/prediction models were created based on PCA-LDA with a maximum number of one tissue type and two animals (e.g., cow [liver] vs. chicken [liver]; Fig. 3) analyzed with both the CO_2_ laser and the diathermic knife. We limited these models to one tissue type and two animals in order to have a total number of four variables in the score plot. From the loading plot of generated classification models (based on molecular fingerprints), we searched for similarities in the spectra between the two handpieces for the same tissue type. We focused on characteristics peaks able to discriminate the two tissue types independently from the handpiece used for sampling.

In order to create a unique peak list for the model, an inclusion and an exclusion peak lists were generated:

i. A tissue-related peak lists with peaks that contributed the most to differentiating between tissue types (inclusion list, green component in Fig. 3).
ii. A handpiece-related peak list with peaks that contributed the most to differentiate samples based on the handpieces (exclusion list, red component in Fig. 3).

These two peak lists were compared and combined as follows: peaks from the exclusion list were removed from the inclusion list to create several tissue-specific peak lists, as well as a combined list containing 50 peaks which contributed the most to the separation of the tissue types independently from the handpiece (ESM Table S6). We found that in this specific application, including less or more than 50 peaks reduces the accuracy of the classification rate for every model. As reported in Table 2, the PCA/LDA peak list–based model led to an improvement for almost all the animal comparisons, with the exception of the models that included duck muscle. An improvement from 59 to 71% good classification in the cross-validation when leaving the “laser out” for the classification of the diathermic data was observed when all tissue samples were included. When excluding skin from the model, there was an improvement from 67 to 87%. Additionally, leaving the “diathermic out” led to an improvement from 29 to 56% in the prediction rate with all the tissues included, and an improvement from 55 to 67% when excluding the data acquired from the skin samples (Table 2). This can be attributed to oversampling. During skin sampling with both handpieces, some of the smoke generated actually belonged to the muscle beneath the skin. Additionally, the skin of selected animals had a very heterogeneous composition (e.g., epidermis, derma, fat) while being extremely thin. All these factors increased the variance of the skin data and for these reasons, we decided to exclude this tissue from the classification model.

The data clearly show that the peak list–based model leads to higher classification rates. As observed in the previous paragraph, a larger variation of the data was observed when sampling with the diathermic knife. The introduction of a tissue-specific peak list led to a higher improvement in the classification rate of the model built with the laser data for similar reasons (Fig. 3a/4). The larger variation of the data from the diathermic knife (green data in Fig. 4) caused a higher variance in the predictive model which results in a higher score during the cross-validation with the laser (low data variation, red data in Fig. 4) [41, 42]. Training a model from the data obtained with the laser creates a model with less variance. Validating this last model with the data from the diathermic knife even if more precise and accurate results in an overall lower classification rate.

### Analysis of hard tissues

**Fig. 5:**
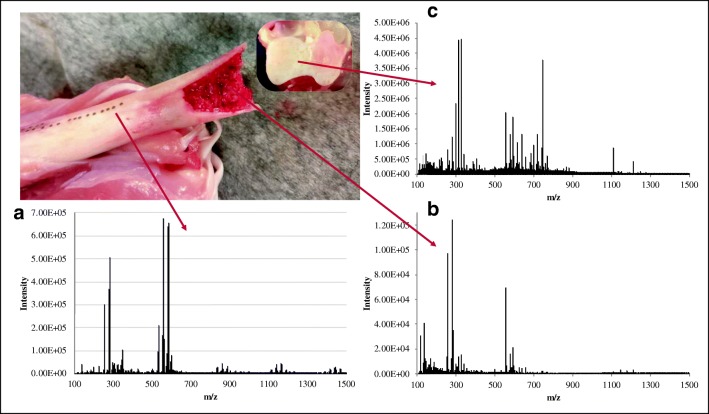
Sampling of hard tissue with the laser-REIMS system. With the CO_2_ laser, it was possible to generate a signal from bone [**a**], bone marrow [**b**], and cartilage [**c**]. It is not possible to generate aerosols and consequently a signal while using the diathermic knife due to the hard nature of the bone

We investigated the possibility of analyzing hard tissues with the laser handpiece. Since malignancies such as soft tissue sarcoma can occur near bones, collecting information from hard tissue in the context of this disease might help with diagnosis and identification of tumor margins. Ultimately this would provide a better understanding and improve outcome of these conditions. Soft tissue sarcomas may rest against the periosteal surface and invade the bone [43–49]. Sampling the bone in vivo with limited invasiveness can be crucial for a better prognosis or choice of therapy [50, 51]. Furthermore, the analysis of hard tissue could also contribute to a better understanding of the healing process in response to bone fractures. Not all bones heal from a fracture and if the fracture occurs near a joint, post-traumatic osteoarthritis can develop [52–54]. The diathermic knife cannot be used to sample dry tissue such as bone or cartilage as it is simply not possible to cut through such tissues with this handpiece due to its physical properties. Furthermore, electrosurgical dissection is based on the joule heating to evaporate of tissues by an electric current [29] which cannot be achieved in tissue that poorly conducts heat, such as bone. Therefore, we assessed the possible use of the CO_2_ laser in acquiring molecular profiles from bone and cartilage. We demonstrated that the laser allowed the generation of lipid-rich smoke from hard tissues like bone and cartilage. Figure 5 displays the spectra generated from bone, cartilage, and bone marrow from a chicken leg. The best results were obtained using a laser power of 25 W for the bone marrow and cartilage and 30 W for the bone with “pulse modality” of 0.3 s and 0.4 s respectively. This indicates that an “ablative” pulse is required for the ejection of aerosols from hard tissues. No signal was obtained with power below 25 W or with the “continuous wave” modality (ESM Fig. S2). Additionally, in order to generate any signal, the continuous wave required an exposure time and power of the laser which caused a complete penetration of the chicken leg. Hence, it is not recommended to use the continuous wave to sample hard tissue. The “super pulse” modality created deeper holes in the tissues without generating any signal. Finally, it is noteworthy that leaving the bone and cartilage inside the biosafety cabinet dried the tissues leading to a decrease of the signal over time. Hence, the ex vivo analysis of hard tissues must be performed in the shortest possible time after collection. We foresee that this will not be a limitation for future in vivo applications.

## Conclusion

This work reports a comparison between a diathermic knife and a CO_2_ laser used as handpieces and coupled to a REIMS-TOF mass spectrometer for instantaneous tissue classification. The laser produced less tissue damage when compared with the diathermic knife. The higher reproducibility of the laser was partly due to the fixed position and controlled ablation time. The laser was able to generate aerosols rich in lipids from hard tissues such as bone, bone marrow, and cartilage tissue, which extends the field of applications of REIMS.

Furthermore, we demonstrated that the data generated with one sampling modality can be employed to classify the data generated with the other handpiece, and vice versa, with the introduction of a model based on peak lists that are tissue-specific and common to the two handpieces. We confirmed that peak-based classification models considerably increase the correct classification rate, in comparison with when only the entire molecular pattern is considered.

Combined, these findings strengthen the value and versatility of REIMS for instantaneous identification/classification of soft and hard tissues and the coupling to different handpieces generating aerosol expands its field of application. We foresee that this approach can be easily translated to clinical applications since the two handpieces used in this work are approved medical devices that are frequently used for surgical procedures.

## Electronic supplementary material

ESM 1 (PDF 1052 kb)
