# Supplementary material for: Tissue classification by rapid evaporative ionization mass spectrometry (REIMS): comparison between a diathermic knife and CO2 laser sampling on classification performance
Source: Anal Bioanal Chem. 2019 Nov 11;411(30):7943–55. doi: 10.1007/s00216-019-02148-8 (PMC6920236; doi:10.1007/s00216-019-02148-8)
Supplement: Supplementary file 1 — (PDF 1052 kb) [file 216_2019_2148_MOESM1_ESM.pdf]

Analytical and Bioanalytical Chemistry

Electronic Supplementary Material

Tissue classification by rapid evaporative ionization mass spectrometry (REIMS): comparison between a diathermic knife and CO_2 laser sampling on classification performance

Michele Genangeli, Ron M.A. Heeren, Tiffany Porta Siegel

Table of content

Figure S1 – Home built laser arm used to maintain the laser in a fixed and safe position during analysis [a]. Control box for the safety arm includes height control to ensure that the laser is at optimal focus position on the tissue analyzed[b]. *3*
Figure S2 – Laser-REIMS spectra obtained from sampling chicken liver with different modalities. CW = continuous wave, Pulsed and super pulse modalities)........ *4*
Figure S3 –Mass spectra obtained from a cow liver sampled using the MALDI-Orbitrap and zoom in the region between m/z 1200-1400. No clusters are visible in the higher mass range........ *5*
Figure S4 – REIMS-LDA-3D score plot including all data acquired from sampling soft tissue types. L=laser. k=diathermic knife........ *6*
Figure S5 - Laser REIMS-LDA-3D score plot including data acquired from sampling soft tissue types but excluding the data related to duck skin. L=laser. k=diathermic knife. *6*
Figure S6 - Laser REIMS-LDA-3D score plot including data acquired from sampling soft tissue types but excluding the data related to duck skin and duck liver. L=laser. k=diathermic knife *7*
Figure S7 - Visualization of bone damage induced by laser sampling using different modalities available with the CO2 laser system. CW = continuous wave........ *8*

Table S1 - Lipid identification from REIMS and MALDI-MS measurements of cow liver tissues. CE= collision energy (eV), Measured REIMS = measured m/z with REIMS-TOF system, Orbitrap = measured m/z with Orbitrap system, Theor.Mass = Theoretical mass (m/z), ppm err = calculated error in part-per-million (for REIMS, Orbitrap), Prod ion = Product ion (m/z, for REIMS), Fragments id = fragments identification, Id = Lipid identification........ 9
Table S2 - Lipid identification from chicken liver measurements. CE= collision energy (eV), Measured REIMS = measured m/z with REIMS-TOF system, Orbitrap = measured m/z with Orbitrap system, Theor.Mass = Theoretical mass (m/z), Ppm err = calculated error in part-per-million (for REIMS, Orbitrap), Prod ion = Product ion (m/z, for REIMS), Fragments id = fragments identification, Id = Lipid identification........ 10
Table S3 - Lipid identification from cow muscle measurements. CE= collision energy (eV), Measured REIMS = measured m/z with REIMS-TOF system, Orbitrap = measured m/z with Orbitrap system, Theor.Mass = Theoretical mass (m/z), Ppm err = calculated error in part-per-million (for REIMS, Orbitrap), Prod ion = Product ion (m/z, for REIMS), Fragments id = fragments identification, Id = Lipid identification........ 11
Table S4 - Lipid identification from chicken muscle measurements. CE= collision energy (eV), Measured REIMS = measured m/z with REIMS-TOF system, Orbitrap = measured m/z with Orbitrap system, Theor.Mass = Theoretical mass (m/z), Ppm err = calculated error in part-per-million (for REIMS, Orbitrap), Prod ion = Product ion (m/z, for REIMS), Fragments id = fragments identification, Id = Lipid identification........ 12
Table S5 – Results from the spectra comparison between the laser and diathermic knife. 13
Table S6 - Peak lists reporting m/z values used to create the different peak list-based models. The m/z values are reported in order of importance for the discrimination of each tissue with the most important values on top. For the “All” model, a combination of peak lists was used accordingly to the tissues included in the model. 14

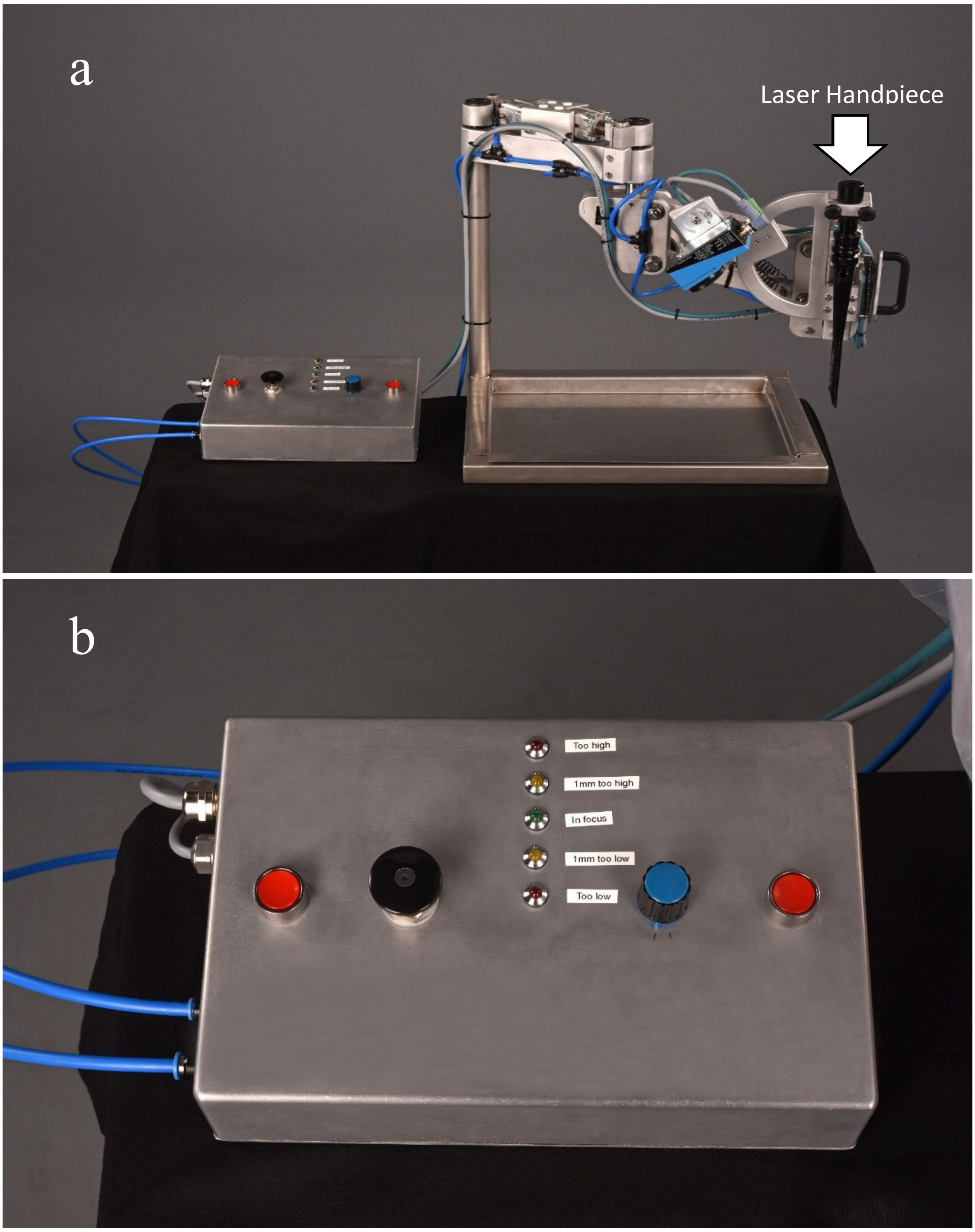

Fig. S1 Home built laser arm used to maintain the laser in a fixed and safe position during analysis [a]. Control box for the safety arm includes height control to ensure that the laser is at optimal focus position on the tissue analyzed [b]

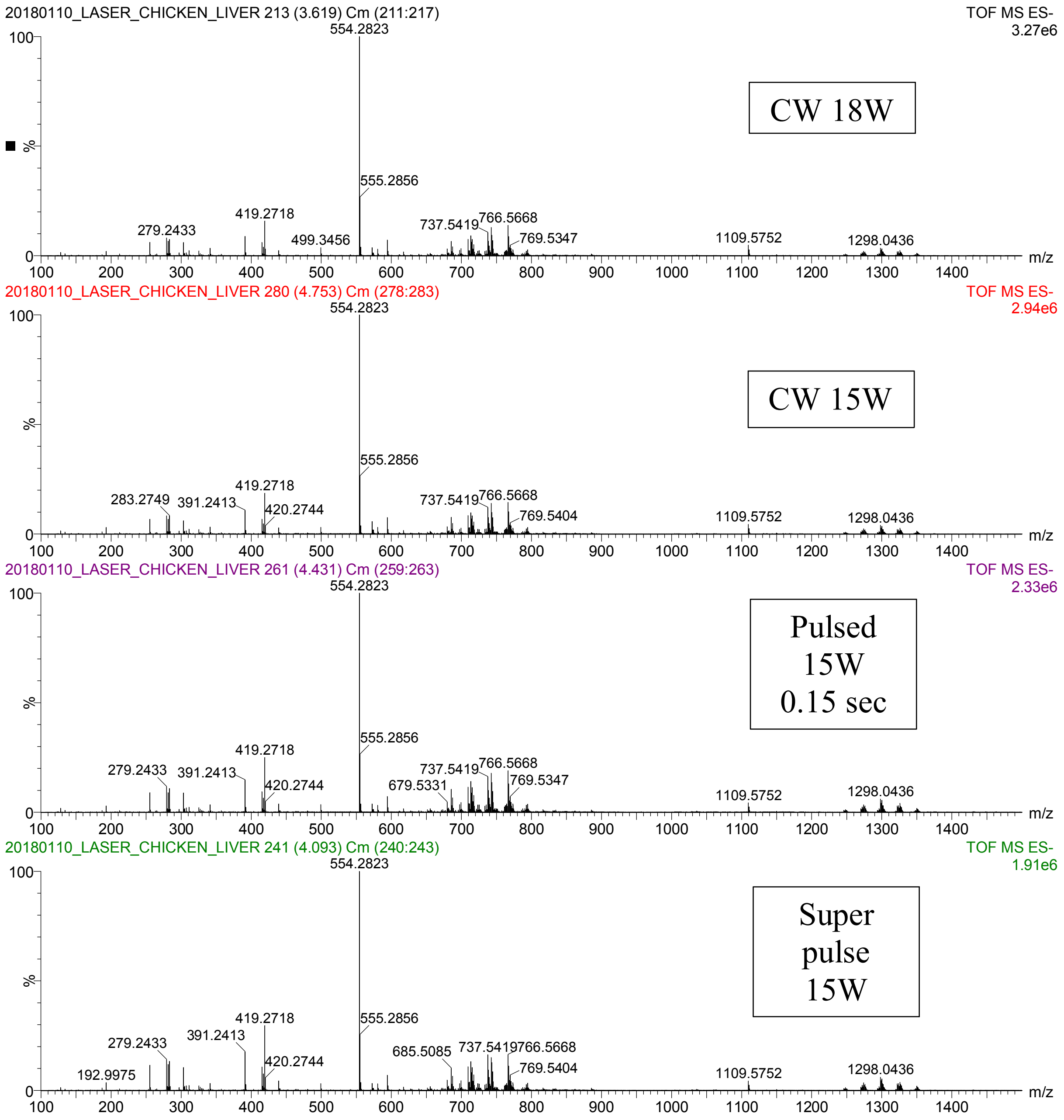

Fig. S2 – Laser-REIMS spectra obtained from sampling chicken liver with different modalities. CW = continuous wave, Pulsed and super pulse modalities

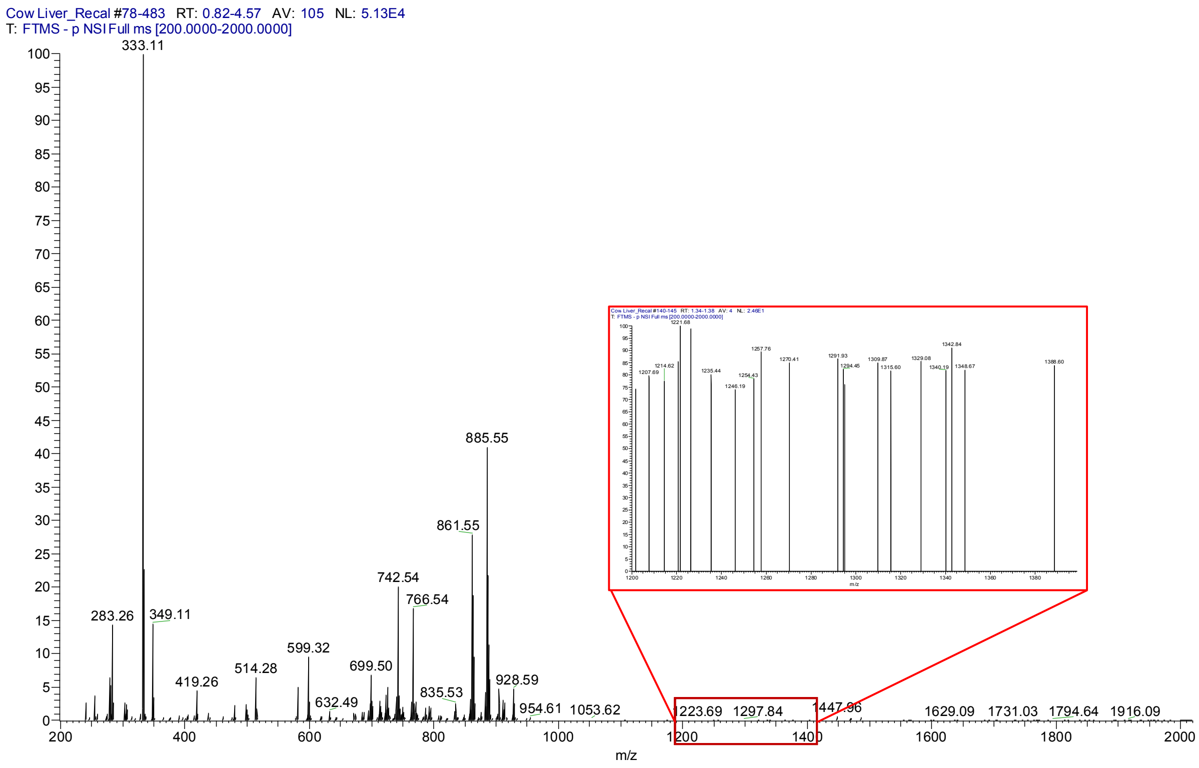

Fig. S3 Mass spectra obtained from a cow liver sampled using the MALDI-Orbitrap and zoom in the region between m/z 1200-1400. No clusters are visible in the higher mass range

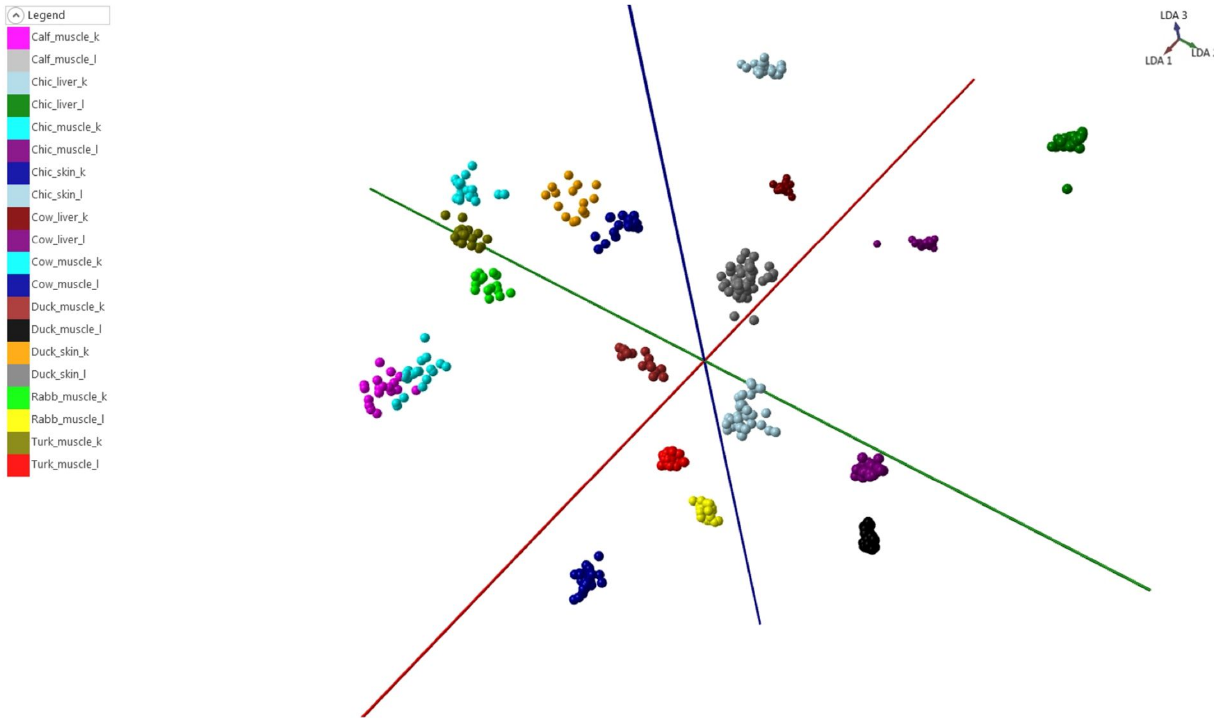

Fig. S4 REIMS-LDA-3D score plot including all data acquired from sampling soft tissue types**.** L=laser. k=diathermic knife

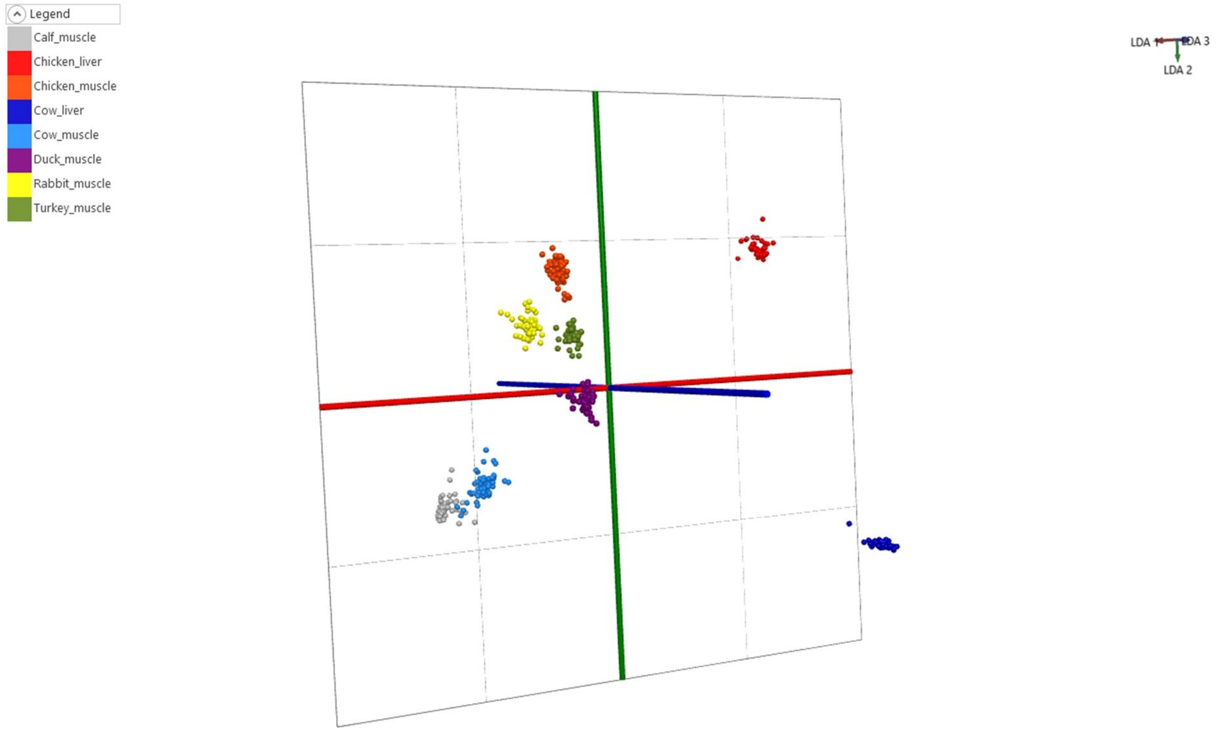

Fig. S5 Laser REIMS-LDA-3D score plot including data acquired from sampling soft tissue types but excluding the data related to duck skin. Laser data only

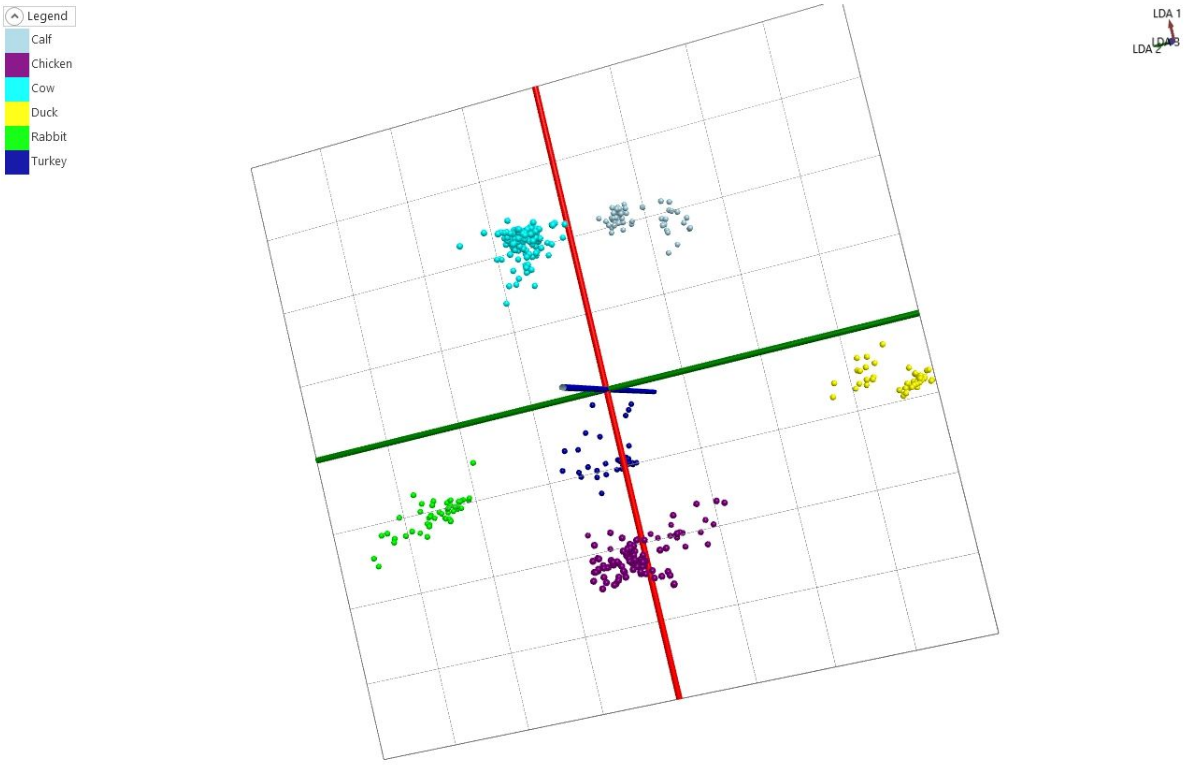

Fig. S6 Laser REIMS-LDA-3D score plot including data acquired from sampling soft tissue types but excluding the data related to duck skin and duck liver. Diathermic data only

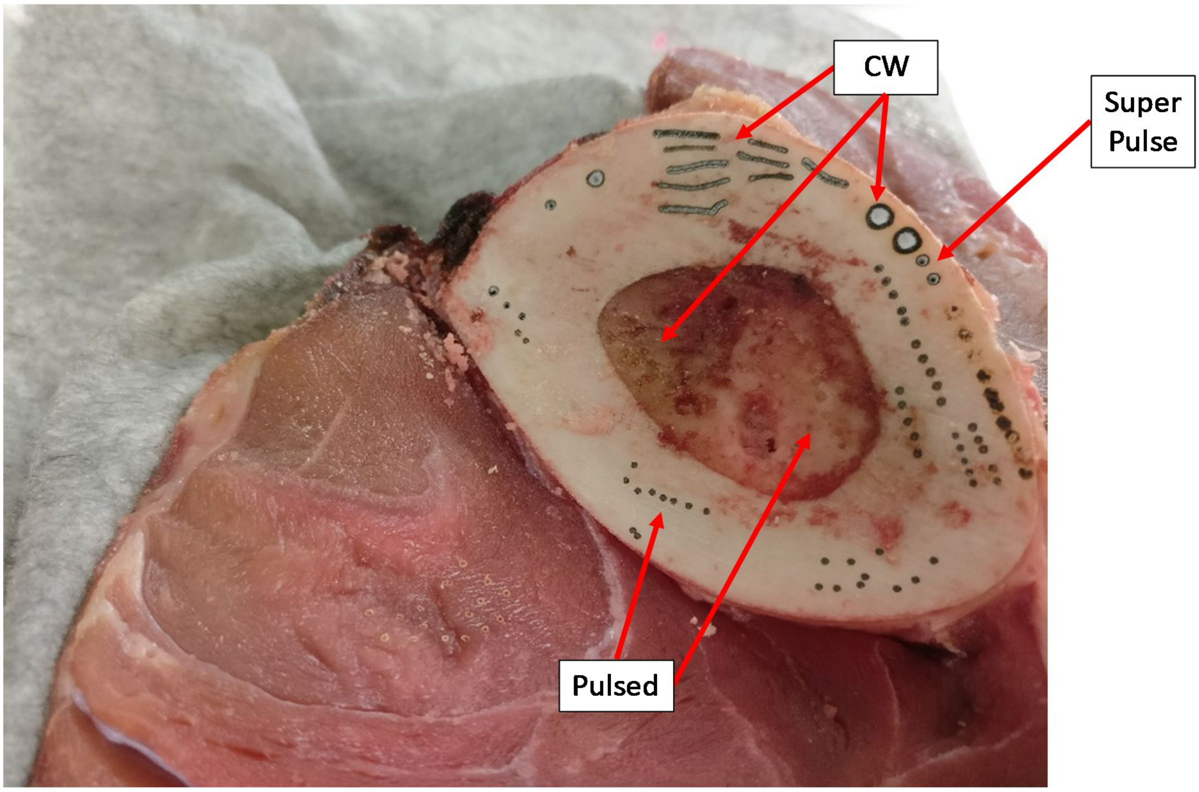

Fig. S7 Visualization of bone damage induced by laser sampling using different modalities available with the CO2 laser system. CW = continuous wave operated in laser dot modality

Table S1 Lipid identification from REIMS and MALDI-MS measurements of cow liver tissues. CE= collision energy (eV), Measured REIMS = measured m/z with REIMS-TOF system, Orbitrap = measured m/z with Orbitrap system, Theor.Mass = Theoretical mass (m/z), ppm err = calculated error in part-per-million (for REIMS, Orbitrap), Prod ion = Product ion (m/z, for REIMS), Fragments id = fragments identification, Id = Lipid identification

	**Cow Liver**			**ppm err**	**ppm err**	**Prod ion**	**Prod ion**	**ppm err**			
**CE Reims**	**Measured REIMS**	**Orbitrap**	**Theor. Mass**	**REIMS**	**Orbitrap**	**REIMS**	**Theor.**	**prod ion**	**fragments id**	**id**	**adduct**
28	713,51	713,5128	713,51286	4,003	0,078	451,28	451,28301	6,679	FA 18:2(-H)		
						283,26	283,26425	15,018	FA 18:0(+O)	Tag 38:2;1	$[M+Cl^-]^-$
						279,23	279,23290	10,386	FA 18:2(+O)		
30	742,52	742,5392	742,53923	25,897	0,039	481,3	481,25719	-88,940	-FA 18:1(-H)	PE 36:2	$[M-H]^-$
						283,26	283,26425	15,018	FA 18:0(+O)		
						279,23	279,23290	10,386	FA 18:2(+O)		
						255,23	255,23295	11,574	FA 16:0(+O)		
30	745,53	745,5025	745,50251	-36,874	0,012	481,3	481,25719	-88,940	-FA 18:1(-H)	PG 34:2	$[M-H]^-$
						283,26	283,26425	15,018	FA 18:0(+O)		
						281,25	281,24860	-4,964	FA 18:1(+O)		
						279,23	279,23290	10,386	FA 18:2(+O)		
						255,23	255,23295	11,574	FA 16:0(+O)		
30	763,5	763,4552	763,45556	-58,207	0,470	451,28	451,28301	6,679	-FA 22:5(-H)	PG 36:7	$[M-H]^-$
						329,25	329,24860	-4,240	FA 22:5(+O)		
						283,26	283,24313	-59,574	FA 22:6(-CO)		
33	793,54	793,5019	793,50251	-47,245	0,767	481,29	481,23606	-112,065	-FA 20:0(+HO) -FA 16:0(-H) -PG(74)	PG 38:6	$[M-H]^-$
						303,23	303,23295	9,742	FA 20:4(+O)		
						283,26	283,26425	15,018	FA 22:6(-CO) - FA 18:0(+O)		
						279,23	279,23290	10,386	FA 18:2(+O)		
						255,23	255,23295	11,574	FA 16:0(+O) - FA 20:6(-CO)		

Table S2 Lipid identification from chicken liver measurements. CE= collision energy (eV), Measured REIMS = measured m/z with REIMS-TOF system, Orbitrap = measured m/z with Orbitrap system, Theor.Mass = Theoretical mass (m/z), Ppm err = calculated error in part-per-million (for REIMS, Orbitrap), Prod ion = Product ion (m/z, for REIMS), Fragments id = fragments identification, Id = Lipid identification

	Chicken Liver			ppm err	ppm err	Prod ion	Prod ion	ppm err			
CE Reims	Measured REIMS	Orbitrap	Theor. Mass	REIMS	Orbitrap	REIMS	Theor.	prod ion	fragments id	id	adduct
28	697,46	697,4812	697,48138	-30,653	-0,25807	435,24	435,25171	26,914	-FA 18:2(-H)		
						417,23	417,24115	26,724	-FA 18:2(+HO)	PA 36:3	$[M-H]^-$
						281,24	281,2486	30,593	FA 18:1(+O)		
						279,22	279,2329	46,200	-FA 28:3(+HO)		
						255,22	255,23295	50,756	FA 16:0(+O)		
30	742,51	742,5392	742,539229	-39,364	-0,03906	480,29	480,30956	40,732	-FA 18:2(-H)	PE 36:2	$[M-H]^-$
						462,28	462,26261	-37,611	-FA 19:0(-H)		
						283,25	283,26425	50,323	FA 18:0(+O)		
						279,22	279,23295	46,394	FA 18:2(+O)		
						255,22	255,23295	50,756	FA 16:0(+O)		
30	766,51	766,539	766,539229	-38,131	-0,29875	480,29	480,30956	40,732	-FA 20:4(-H)		
						462,28	462,299	41,098	FA 20: 4 (-OH)	PE 38:4	$[M-H]^-$
						303,22	303,23295	42,721	FA 20:4(+O)		
						283,25	283,26425	50,323	FA 18:0(+O)		
						255,22	255,23295	50,756	FA 16:0(+O)		
30	767,5	767,4865	767,486859	17,122	-0,46776	577,23	577,42387	335,854	-FA 12:5(+HO)	PG 36:5	$[M-H]^-$
						555,25	555,34562	172,202	-FA 9:1(-H) -PG(74)		
						481,28	481,25719	-47,388	-FA 20:4(-H)		
						303,22	303,23295	42,721	FA 20:4(+O)		
						283,26	283,26425	15,018	FA 18:0(+O)		
						255,22	255,23295	50,756	FA 16:0(+O)		
30	773,51	773,5336	773,533809	-30,78	-0,27019	491,26	491,27793	36,496	-FA 1:18(+HO) / -FA 1:14(-H) -PG(74)	PG 36:2	$[M-H]^-$
						417,23	417,24115	26,724	-FA 18:1(+HO) -PG(74)		
						307,25	307,26425	46,392	FA 20:2(+O)		

						279,22	279,23295	46,394	FA 18:2(+O)		
						255,22	255,23295	50,756	FA 14:1(+O) / FA 16:0(+O)		
						152,99	152,99583	38,133	PG(153)		

Table S3 Lipid identification from cow muscle measurements. CE= collision energy (eV), Measured REIMS = measured m/z with REIMS-TOF system, Orbitrap = measured m/z with Orbitrap system, Theor.Mass = Theoretical mass (m/z), Ppm err = calculated error in part-per-million (for REIMS, Orbitrap), Prod ion = Product ion (m/z, for REIMS), Fragments id = fragments identification, Id = Lipid identification

	Cow Muscle			ppm err	ppm err	Prod ion	Prod ion	ppm err			
CE Reims	Measured REIMS	Orbitrap	Theor. Mass	REIMS	Orbitrap	REIMS	Theor.	prod ion	fragments id	id	adduct
28	681,45	681,3777	681,37731	-106,7	-0,574	419,25	419,25680	16,219	-FA 12:6(+HO) -PG(74)	PG 30:6	$[M-H]^-$
						401,18	401,17347	-16,289	-FA 14:2(-H) -PG(74)		
						375,20	375,15782	-112,433	-FA 16:3(-H) -PG(74)		
						279,23	279,23295	10,579	FA 18:2(+O)		
						253,21	253,21730	28,846	FA 16:1(+O)		
30	715,55	715,4559	715,45556	-132,0	-0,477	283,25	283,26425	50,323	FA 18:0(+O)	PG 32:3	$[M-H]^-$
						281,24	281,24860	30,593	FA 18:1(+O)		
						279,22	279,23295	46,394	FA 18:2(+O)		
						255,22	255,23295	50,756	FA 16:0(+O)		
30	717,48	717,4716	717,47121	-12,253	-0,545	453,25	453,22589	-53,187	-FA 18:1(-H)		
						281,24	281,24860	30,593	FA 18:1(+O)	PG 32:2	$[M-H]^-$
						255,22	255,23295	50,756	FA 16:0(+O)		
						253,21	253,21730	28,846	FA 16:1(+O)		
30	750,51	750,5441	750,54546	47,244	1,808	464,30	464,31465	31,549	-FA 20:4(-H)		
						436,27	436,28335	30,596	-FA 22:4(-H)	PE O-38:5	$[M-H]^-$
						331,25	330,14758	-3328,051	-FA 28:2(+HO)		
						303,22	303,23295	42,721	FA 20:4(+O)		
						283,26	283,26425	15,018	FA 18:0(+O)		
						196,03	195,13905	-4544,952	FA 12:2(+O)		

33	863,54	863,5659	863,56550	29,533	-0,460	581,29	581,30962	33,758	-FA 18:1(+HO)	PI 36:1 (18:0/18:1)	$[M-H]^-$
						419,25	419,25680	16,219	FA 18:0(+C3H5O5P)		
						297,03	297,11086	272,242	FA 8:0(+C3H7O6P)		
						283,25	283,26425	50,323	FA 18:0(+O)		
						281,24	281,24860	30,593	FA 18:1(+O)		
						259,01	259,02244	48,041	PI(259)		
						241,00	241,01188	49,286	PI(241)		
						223,00	223,00131	5,888	PI(223)		

Table S4 Lipid identification from chicken muscle measurements. CE= collision energy (eV), Measured REIMS = measured m/z with REIMS-TOF system, Orbitrap = measured m/z with Orbitrap system, Theor.Mass = Theoretical mass (m/z), Ppm err = calculated error in part-per-million (for REIMS, Orbitrap), Prod ion = Product ion (m/z, for REIMS), Fragments id = fragments identification, Id = Lipid identification

	Chicken Muscle			ppm err	ppm err	Prod ion	Prod ion	ppm err			
**CE Reims**	**Measured REIMS**	**Orbitrap**	**Theor. Mass**	**REIMS**	**Orbitrap**	**REIMS**	**Theor.**	**prod ion**	**fragments id**	**id**	**adduct**
28	697,46	697,4812	697,48138	-30,653	-0,258	435,24	435,25171	26,914	-FA 18:2(-H)		
						281,24	281,24860	30,593	FA 18:1(+O)	PA 36:3	$[M-H]^-$
						279,22	279,23295	46,394	FA 18:2(+O)		
						255,22	255,23295	50,756	FA 16:0(+O)		
28	699,47	699,4967	699,49703	-38,642	-0,472	435,23	435,25171	49,891	-FA 18:1(-H)		
						419,24	419,25680	40,073	-FA 18:2(+HO)	PA 36:2	$[M-H]^-$
						281,24	281,24860	30,593	FA 18:1(+O)		
						255,22	255,23295	50,756	FA 16:0(+O)		
30	766,51	766,539	766,53923	-38,131	-0,299	480,29	480,30956	40,732	-FA 20:4(-H)		
						303,22	303,23295	42,721	FA 20:4(+O)	PE (18:0/20:4)	$[M-H]^-$
						283,25	283,26425	50,323	FA 18:0(+O)		
						255,22	255,23295	50,756	FA 16:0(+O)		
33	885,53	885,5498	885,54985	-22,419	-0,060	599,31	599,32019	16,998	-FA 20:4(-H)	PI(20:4/18:0)	$[M-H]^-$

						581,29	581,30962	33,758	-FA 20:4(+HO)		
						419,24	419,25680	40,073	FA 18:0(+C3H5O5P)		
						303,22	303,23295	42,721	FA 20:4(+O)		
						283,26	283,26425	15,018	FA 18:0(+O)		
						259,01	259,02244	48,041	PI(259)		
						241,00	241,01188	49,286	PI(241)		
						223,00	223,00131	5,888	PI(223)		

Table S5 Results from the spectra comparison between the laser and diathermic knife

	**TIC**	**Noise level**	**S/N**	**n peaks detectable**	**Avg. intensity n=10 peaks**
	(n=10), CV	n=10	s/n, (n=10)		n=5 consecutive burns, CV
Laser	11,31%	-	+ 1,7x	+ 7%*	0,76%
Diathermic	15,25%	1,6x	-		1,94%

*more peaks detectable due to lipid clusters

Table S6 Peak lists reporting m/z values used to create the different peak list-based models. The m/z values are reported in order of importance for the discrimination of each tissue with the most important values on top. For the "All" model, a combination of peak lists was used accordingly to the tissues included in the model

Muscle						Liver	
cow	calf	chicken	duck	turkey	rabbit	cow	chicken
717,50	742,50	676,50	636,50	881,50	874,50	897,50	620,50
622,50	697,50	855,50	878,50	699,50	824,50	754,50	646,50
611,50	743,50	700,50	739,50	748,50	885,50	607,50	621,50
792,50	726,50	701,50	621,50	747,50	718,50	713,50	766,50
721,50	698,50	744,50	769,50	666,50	886,50	793,50	773,50
705,50	715,50	728,50	709,50	743,50	691,50	743,50	697,50
764,50	699,50	703,50	697,50	725,50	744,50	715,50	742,50
718,50	725,50	730,50	770,50	723,50	769,50	741,50	767,50
612,50	727,50	702,50	699,50	612,50	794,50	711,50	738,50
776,50	770,50	750,50	747,50	697,50	750,50	745,50	709,50
719,50	681,50	671,50	775,50	714,50	752,50	763,50	721,50
765,50	752,50	729,50	771,50	722,50	887,50	765,50	774,50
720,50	768,50	745,50	695,50	726,50	715,50	863,50	762,50
793,50	741,50	886,50	723,50	701,50	778,50	735,50	699,50
790,50	724,50	719,50	725,50	683,50	751,50	861,50	698,50
749,50	744,50	691,50	751,50	749,50	745,50	701,50	611,50
818,50	751,50	704,50	710,50	724,50	719,50	820,50	733,50
623,50	716,50	683,50	713,50	770,50	670,50	714,50	679,50
693,50	700,50	715,50	766,50	741,50	730,50	712,50	612,50
737,50	685,50	731,50	711,50	773,50	795,50	775,50	722,50
701,50	753,50	672,50	743,50	775,50	673,50	887,50	725,50
644,50	771,50	722,50	792,50	772,50	780,50	746,50	790,50
767,50	754,50	716,50	834,50	768,50	779,50	796,50	771,50
735,50	769,50	726,50	772,50	727,50	753,50	772,50	710,50
731,50	740,50	657,50	748,50	681,50	700,50	770,50	642,50
791,50	683,50	687,50	767,50	713,50	703,50	821,50	685,50
777,50	709,50	629,50	698,50	740,50	674,50	883,50	695,50
816,50	671,50	642,50	749,50	774,50	861,50	792,50	750,50
650,50	887,50	780,50	724,50	667,50	766,50	736,50	749,50
600,50	682,50	741,50	696,50	623,50	792,50	716,50	834,50
834,50	861,50	684,50	737,50	738,50	692,50	727,50	687,50
706,50	747,50	778,50	794,50	657,50	739,50	888,50	726,50
819,50	723,50	661,50	863,50	787,50	888,50	795,50	787,50
883,50	728,50	893,50	833,50	789,50	704,50	776,50	673,50
613,50	686,50	746,50	835,50	655,50	868,50	864,50	622,50
758,50	862,50	891,50	707,50	815,50	781,50	889,50	737,50
820,50	755,50	679,50	832,50	810,50	734,50	862,50	835,50
763,50	672,50	779,50	831,50	639,50	675,50	729,50	682,50
654,50	889,50	756,50	789,50	684,50	831,50	816,50	700,50
614,50	669,50	867,50	864,50	664,50	601,50	777,50	739,50
804,50	822,50	754,50	787,50	679,50	702,50	702,50	785,50
602,50	756,50	689,50	714,50	696,50	659,50	822,50	788,50
648,50	823,50	659,50	838,50	640,50	844,50	859,50	885,50
817,50	814,50	692,50	841,50	651,50	618,50	791,50	680,50
680,50	825,50	670,50	773,50	838,50	797,50	748,50	686,50
607,50	798,50	630,50	795,50	634,50	647,50	808,50	734,50
609,50	712,50	658,50	850,50	638,50	867,50	818,50	833,50
665,50	826,50	631,50	733,50	656,50	661,50	817,50	666,50
